# Enhancing the Anticancer Activity of a Carcinoma-Directed Peptide–HLA-I Fusion Protein by Armoring with Mutein IFNα

**DOI:** 10.3390/ijms26073178

**Published:** 2025-03-29

**Authors:** Douwe Freerk Samplonius, Anne Paulien van Wijngaarden, Lisanne Koll, Xiurong Ke, Wijnand Helfrich

**Affiliations:** University of Groningen, Laboratory for Translational Surgical Oncology, Department of Surgery, University Medical Center Groningen, 9713 GZ Groningen, The Netherlands; d.f.samplonius@umcg.nl (D.F.S.); a.p.van.wijngaarden@umcg.nl (A.P.v.W.); l.koll@umcg.nl (L.K.); x.ke@umcg.nl (X.K.)

**Keywords:** immunotherapy, cancer, Merkel cell carcinoma, antiviral immunity, cytokines

## Abstract

Previously, we reported on the peptide–HLA-I fusion protein EpCAM-ReTARG^TPR^, which allows us to redirect the cytotoxic activity of pre-existing anti-CMV CD8^pos^ T cell immunity to selectively eliminate EpCAM^pos^ cancer cells. EpCAM-ReTARG^TPR^ consists of the CMV pp65-derived peptide TPRVTGGGAM (TPR) fused in tandem with a soluble HLA-B*07:02/β2-microglobulin (β2M) molecule and an EpCAM-directed Fab antibody fragment. To further enhance its anticancer activity, we equipped EpCAM-ReTARG^TPR^ with the immune-potentiating cytokine muteins IL2^(H16A,F42A)^ and IFNα^R149A^, respectively. Both cytokines are engineered to have attenuated affinity for their respective cytokine receptors. Compared to EpCAM-ReTARG^TPR^, in vitro treatment of EpCAM^pos^ carcinoma cell lines with EpCAM-ReTARG^TPR^vIL2 for 24 h increased the cytotoxic activity of PBMCs containing low levels of TPR-specific CD8^pos^ T cells by ~15%, whereas EpCAM-ReTARG^TPR^IFNα^R149A^ induced an increase of ~50%. Moreover, treatment for 120 h with EpCAM-ReTARG^TPR^IFNα^R149A^ inhibited the proliferative capacity of the cancer cell lines OvCAR3 and PC3M by ~91% without compromising the viability of the TPR-specific CD8^pos^ T cells and increased their capacity for IFNγ secretion. Importantly, EpCAM-ReTARG^TPR^IFNα^R149A^ potently induced the elimination of primary EpCAM^pos^ refractory carcinoma cells from a Merkel cell carcinoma (MCC) patient. Taken together, the armoring of the carcinoma-directed peptide–HLA-I fusion protein EpCAM-ReTARG^TPR^ with IFNα^R149A^ potently enhanced the efficacy of pre-existing anti-CMV CD8^pos^ T cell immunity to selectively eliminate EpCAM^pos^ cancer cells.

## 1. Introduction

Typically, cytomegalovirus (CMV)-seropositive individuals harbor a high percentage of CMV-specific CD8^pos^ T cells directed against immunodominant viral peptides, which may increase with age up to 20% [1]. These so-called ‘inflationary’ anti-CMV CD8^pos^ T cells possess an effector–memory phenotype, maintain a ready-to-go functional status, and exhibit the capacity to migrate into virtually all tissues, including tumor tissues [2]. In recent years, several strategies that aim to redirect the unique cytotoxic potential of inflationary anti-CMV CD8^pos^ T cells to selectively eliminate cancer cells have been developed (reviewed in [3]).

Previously, we reported on a novel Fab–peptide–HLA-I fusion protein, designated EpCAM-ReTARG^TPR^, that consists of an EpCAM-directed Fab antibody fragment fused to HLA-B*07:02-β2-microglobulin (β2M) and genetically equipped with the CMV pp65-derived peptide TPRVTGGGAM (TPR). EpCAM-ReTARG^TPR^ showed potent in vitro capacity to redirect TPR-specific CD8^pos^ T cells derived from CMV-seropositive/HLA-B*07:02^pos^ individuals to eliminate various types of EpCAM-expressing carcinomas [4]. The ReTARG approach selectively engages the TCR-CD3 signaling complex of cognate CMV-specific CD8^+^ T cells in a physiologically normal manner, effectively eliminating EpCAM^pos^ cancer cells without excessive cytokine release. In contrast, CD3-based T-cell engagers like the BiTE solitomab activate all CD3^pos^ T cells, including inhibitory CD4^pos^ Tregs, by stimulating the CD3ε chain regardless of T-cell specificity. This non-physiological hyperactivation is strongly associated with massive cytokine release and immune-related side effects.

Previously, we compared EpCAM-ReTARG^TPR^ and solitomab in terms of cancer cell elimination and T-cell-secreted cytokines. Our results confirmed that EpCAM-ReTARG^TPR^ effectively engaged CMV-specific CD8^pos^ T cells to eliminate EpCAM^pos^ cancer cells with potency comparable to solitomab, without inducing excessive cytokine release.

Building upon our findings, in the present work, we aimed to further enhance the anticancer activity of the EpCAM-ReTARG^TPR^ approach by genetically equipping this fusion protein with the immune-potentiating cytokine muteins interleukin-2 (IL2) and IFNα-2b, respectively.

IL2 is a pleiotropic cytokine which acts via low-, intermediate-, and high-affinity IL2 receptors (IL2Rs), which are differentially expressed on various types of immune cells. CD25 (also known as IL2Rα) acts as the low-affinity IL2R. The intermediate-affinity IL2R is composed of CD122 (IL2Rβ) and CD132, which are typically expressed on resting immune cells. However, upon TCR-mediated activation, CD25 is rapidly co-expressed and binds with low affinity to IL2, which results in the formation of the heterotrimeric high-affinity IL2R by engaging with CD122 and CD132 (2R) [5]. In contrast, highly immunosuppressive intratumoral regulatory T cells (T_regs_) constitutively express high levels of CD25, which leads to enhanced cell surface levels of the high-affinity IL2R, thereby depleting IL2 that is essential for the expansion and activity of neighboring anticancer T cells [6,7]. Therefore, engineered variant forms of IL2 have been developed with attenuated binding affinity for CD25. In particular, IL2, containing the amino acid mutations H16A and F42A (hereafter referred to as vIL2), has shown to have >100-fold reduced affinity for CD25, which reduces the bias towards T_reg_ activation and selectively promotes the expansion of CD8^pos^ effector T cells [8,9,10]. We therefore reasoned that arming EpCAM-ReTARG^TPR^ with vIL2 may enhance its anticancer capacity in an EpCAM-directed manner. To this end, we constructed EpCAM-ReTARG^TPR^vIL2 in which two linker-interspersed copies of mutein vIL2 were genetically fused to the constant domain of the light chain (CL) of the anti-EpCAM Fab fragment.

Analogously, we constructed EpCAM-ReTARG ^TPR^IFNα^R149A^ in which mutein IFNα^R149A^ is genetically fused to the CL domain of the anti-EpCAM Fab fragment. The pleiotropic activity of IFNα appears to be of particular promise given its potent dual anticancer and immune-potentiating activities. Like all type 1 IFN family members, IFNα binds to the interferon-α/β receptor (IFNAR), which is composed of two subunits, namely IFNAR1 and IFNAR2. Various recombinant IFNα formulations have been employed for a wide range of indications, including cancer, although their efficacy is hampered by dose-limiting side effects as a result of IFNAR expression on essentially all nucleated cells. It was previously shown that the fusion of IFNα mutein IFNα ^R149A^, with a 200-fold reduced affinity for the IFNAR2, to a cancer-directed antibody is less likely to bind to IFNAR expressed on normal cells, thus potentially reducing deleterious off-cancer side effects while ‘en route’ to cancer cells [11,12]. Importantly, antibody-mediated binding to the cancer cell surface locally increases the concentration of IFNα ^R149A^ such that this compensates for its reduced capacity to bind and activate IFNAR1/2. Since IFNAR1/2 molecules are present on both cancer cells and neighboring tumor-infiltrating immune cells, dual anticancer and immune-potentiating activities are potentially achieved in a tumor-directed manner. Therefore, we reasoned that it may be feasible to exploit these favorable characteristics by genetically equipping EpCAM-ReTARG^TPR^ with the IFNα^R149A^ domain.

Here, we investigated the effects of arming peptide–HLA-I fusion proteins with potentiating cytokines and assessed whether their antiproliferative and immune-potentiating activities resulted in overall enhanced anticancer capacity.

## 2. Results

### 2.1. EpCAM-ReTARG^TPR^vIL2 and EpCAM-ReTARG^TPR^IFNα^R149A^ Selectively Bind to EpCAM^pos^ Cancer Cells, Whereupon Their Respective Cytokine Muteins Attain Enhanced Biological Activity

**Figure 1:**
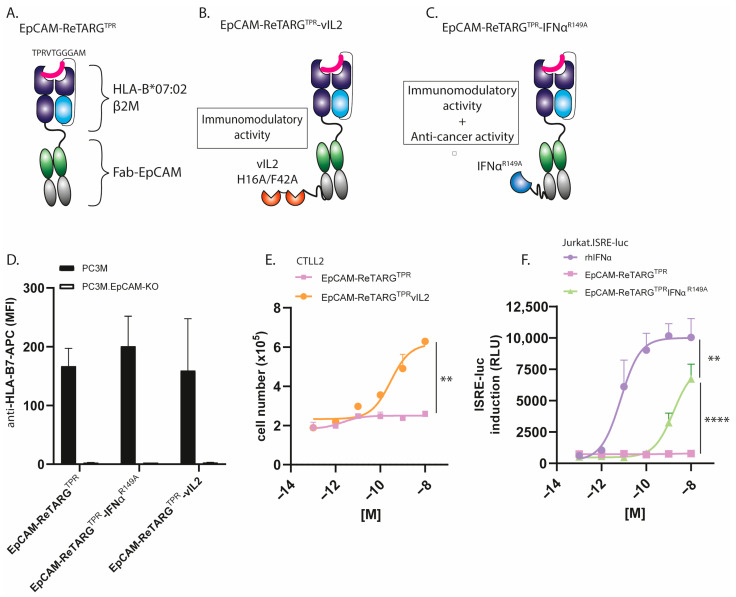
EpCAM-ReTARG^TPR^vIL2 and EpCAM-ReTARG^TPR^IFNα^R149A^ selectively bind to EpCAM^pos^ cancer cells, whereupon their respective cytokine muteins attain enhanced biological activity. (**A**–**C**) Schematic representation of the (**A**) EpCAM-ReTARG^TPR^, (**B**) EpCAM-ReTARG^TPR^vIL2, and (**C**) EpCAM-ReTARG^TPR^IFNα^R149A^ fusion proteins. The mode of action is shown Figure A1 (**D**) Binding of EpCAM-ReTARG^TPR^ fusion proteins (each 10 nM) to EpCAM^pos^ PC3M vs. PC3M.EpCAM-KO cancer cells. (**E**) A proliferation assay of IL2-dependent murine CTLL2 cells in the presence of increasing concentrations of EpCAM-ReTARG^TPR^vIL2 and EpCAM-ReTARG^TPR^, respectively. (**F**) Dose-dependent activation of ISRE in EpCAM^neg^ Jurkat.ISRE-luc reporter cells cultured in the presence of increasing concentrations (0.001–10 nM) of rhIFNα, EpCAM-ReTARG^TPR^, and EpCAM-ReTARG^TPR^IFNα^R149A^, respectively. Statistical analysis was performed using unpaired *t*-test (**E**) and one-way ANOVA (**F**). (** *p* < 0.01 and **** *p* < 0.0001).

**Figure A1:**
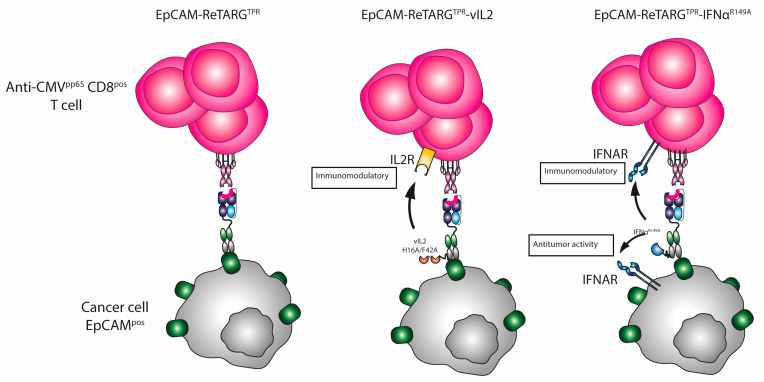
The mode of action of EpCAM-ReTARG^TPR^, EpCAM-ReTARG^TPR^vIL2, and EpCAM-ReTARG^TPR^IFNα^R149A^.

**Figure A2:**
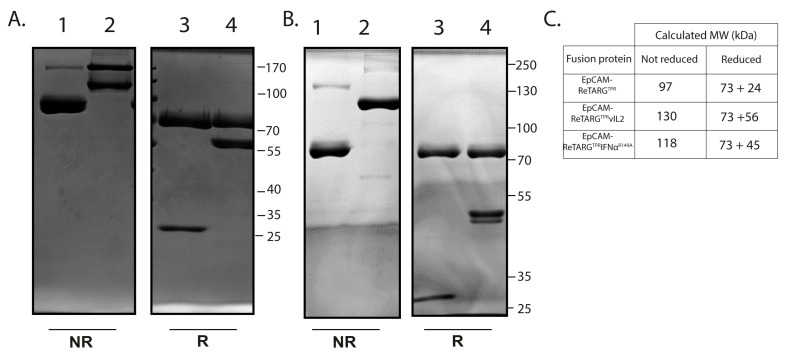
SDS-PAGE analysis of EpCAM-ReTARG^TPR^, EpCAM-ReTARG^TPR^vIL2, and EpCAM-ReTARG^TPR^IFNα^R149A^. (**A**) SDS-PAGE analysis with Coomassie brilliant blue staining of EpCAM-ReTARG^TPR^ (lane 1,3) and EpCAM-ReTARG^TPR^vIL2 (lane 2,4) under non-reducing (NR, lane 1,2) and reducing (R, lane 3,4) conditions. M  =  marker. (**B**) SDS-PAGE analysis with Coomassie brilliant blue staining of EpCAM-ReTARG^TPR^ (lane 1,3) and EpCAM-ReTARG^TPR^IFNα^R149A^ (lane 2,4) under non-reducing (NR, lane 1,2) and reducing (R, lane 3,4) conditions. M  =  marker. (**C**) Calculated MWs of EpCAM-ReTARG^TPR^, EpCAM-ReTARG^TPR^vIL2, and EpCAM-ReTARG^TPR^IFNα^R149A^ in NR and R conditions.

**Figure A3:**
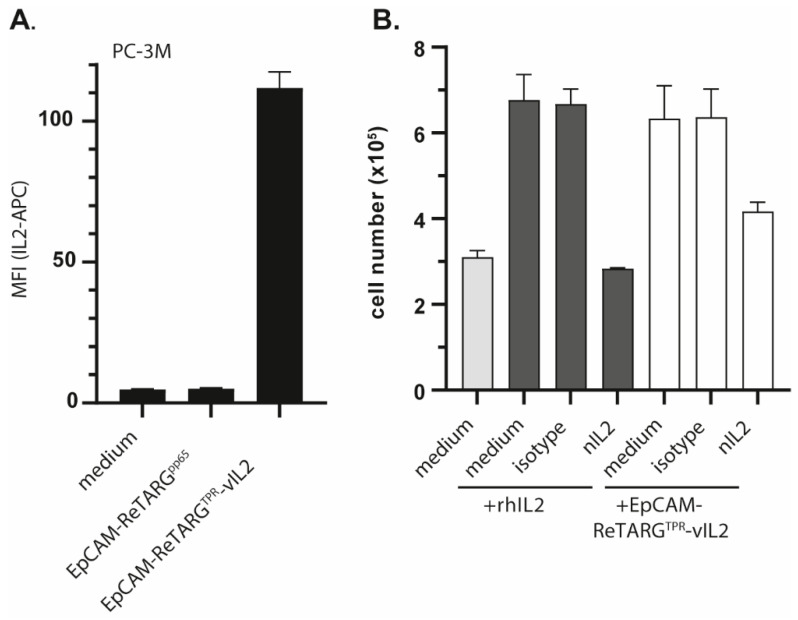
EpCAM-ReTARG^TPR^vIL2 retains IL2R signaling activity. (**A**) Binding of APC-labeled IL2 antibody (2 µg/mL) to PC-3M cancer cells pre-incubated (or not) with EpCAM-ReTARG^TPR^vIL2 or EpCAM-ReTARG^TPR^ (both 10 nM). (**B**) A proliferation assay of human IL2-dependent murine CTLL2 cells in the presence of rh IL2 (12.5 IU/mL) and EpCAM-ReTARG^TPR^vIL2 (2.5 nM), respectively. Additionally, rh IL2 and EpCAM-ReTARG^TPR^vIL2 were pre-incubated with the nIL2 antibody or an appropriate isotype control (both 0.5 µg/mL). Graphs were assessed via flow cytometry and show representative images with two technical replicates (mean ± SD).

**Figure A4:**
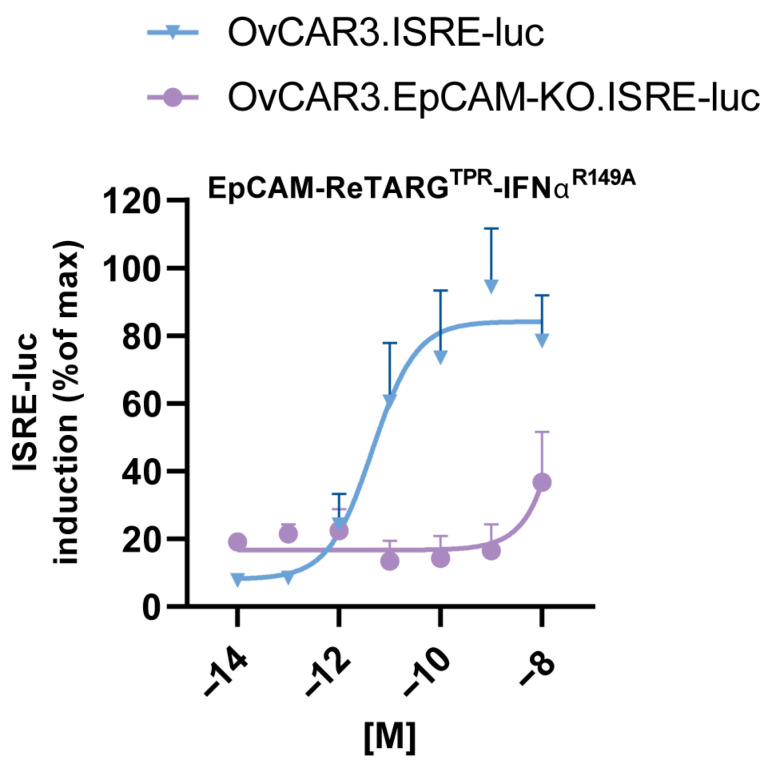
EpCAM-ReTARG^TPR^IFNα^R149A^ fusion protein induces potent EpCAM-directed IFNα activity. Dose-dependent ISRE activation in EpCAM^pos^ OvCAR3.ISRE-luc or EpCAM-KO OvCAR3.ISRE-luc reporter cells incubated with increasing concentrations of EpCAM-ReTARG^TPR^IFNα^R149A^. *n*  =  3 (two technical replicates); means  ±  SDs are shown.

To increase the anticancer activity of EpCAM-ReTARG^TPR^, we designed and constructed EpCAM-ReTARG^TPR^vIL2 and EpCAM-ReTARG^TPR^IFNα^R149A^ (Figure 1A–C, mode-of-action Figure A1). SDS-PAGE analysis of purified EpCAM-ReTARG^TPR^, EpCAM-ReTARG^TPR^vIL2, and EpCAM-ReTARG^TPR^IFNα^R149A^ indicated that their respective apparent molecular weights (MWs) were essentially as calculated (Figure A2). EpCAM-ReTARG^TPR^, EpCAM-ReTARG^TPR^vIL2, and EpCAM-ReTARG^TPR^IFNα^R149A^ selectively bound to EpCAM-expressing PC3M cancer cells with no detectable binding to PC3M.EpCAM-KO cells (Figure 1D). EpCAM-ReTARG^TPR^vIL2 dose-dependently induced the proliferation of the IL2-dependent cell line CTLL2, whereas EpCAM-ReTARG^TPR^ failed to do so (Figure 1E and Figure A3). EpCAM-ReTARG^TPR^vIL2-induced proliferation of CTLL2 cells was inhibited when incubated in the presence of a surplus of an IL2-neutralizing antibody (Figure A3). IFNα-mediated biological activity of EpCAM-ReTARG^TPR^IFNα^R149A^ was confirmed using ISRE-luc transduced Jurkat reporter cells, which showed dose-dependent activation (Figure 1F). Furthermore, the incubation of ISRE-luc-transduced OvCAR3 and OvCAR3.EpCAM-KO cells with increasing concentrations of EpCAM-ReTARG^TPR^IFNα^R149A^ (0.001–10 nM) resulted in potent dose-dependent activation of ISRE in OvCAR3.ISRE-luc cells, whereas the activation in OvCAR3.EpCAM-KO cells was absent, indicating that EpCAM-ReTARG^TPR^IFNα^R149A^ attains IFNα activity in a strict EpCAM-dependent manner (Figure A4). Taken together, our data demonstrate that both EpCAM-ReTARG^TPR^vIL2 and EpCAM-ReTARG^TPR^IFNα^R149A^ selectively bind to EpCAM^pos^ cancer cells, whereupon their respective cytokine muteins attain enhanced biological activity.

### 2.2. Ex Vivo-Expanded TPR-Specific CD8^pos^ T Cells Are Capable of Target Cell Lysis

**Figure 2:**
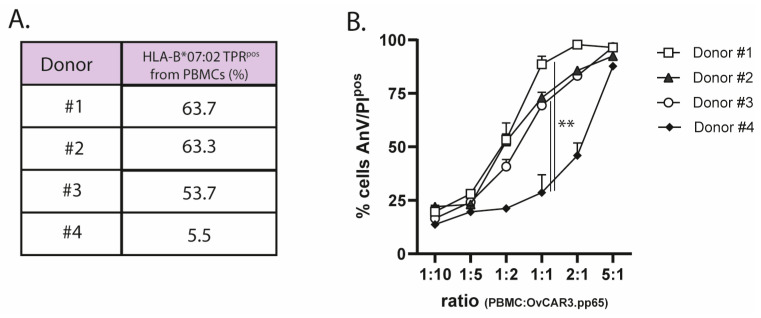
Ex vivo-expanded TPR-specific CD8^pos^ T cells are capable of target cell lysis. (**A**) The percentage of HLA-B*07:02-restricted TPR-specific CD8^pos^ T cells was analyzed using streptamer staining via flow cytometry. (**B**) The cytotoxic capacity of expanded effector cells from 4 donors towards OvCAR3.pp65 cells at the indicated E:T (PBMC:OvCAR.pp65) cell ratios. Apoptotic cancer cell death was assessed using Annexin-V/PI staining after 24 h. Statistical analysis was performed using one-way ANOVA. (** *p* < 0.01).

PBMCs from four HLA-B*07:02^pos^/CMV-seropositive individuals were subjected to a standard ex vivo-expansion protocol (TPR peptide + IL2), after which the percentages of TPR-specific CD8^pos^ T cells were quantified through flow cytometry with streptamer staining (Figure 2A). The TPR/IL2-expanded PBMC effector cells, at incremental effector-to-target (E:T) cell ratios, showed potent capacity for eliminating OvCAR3.pp65 target cells. As expected, TPR-specific CD8^pos^ T cells expanded from donor #4 induced significantly less cancer cell lysis than those from donors #1, #2, and #3 (Figure 2B).

### 2.3. EpCAM-ReTARG^TPR^IFNα^R149A^ Potently Enhances the Cytotoxic Capacity of Ex Vivo-Expanded TPR-Specific CD8^pos^ T Cells and Retains EpCAM-Selective Activity

**Figure 3:**
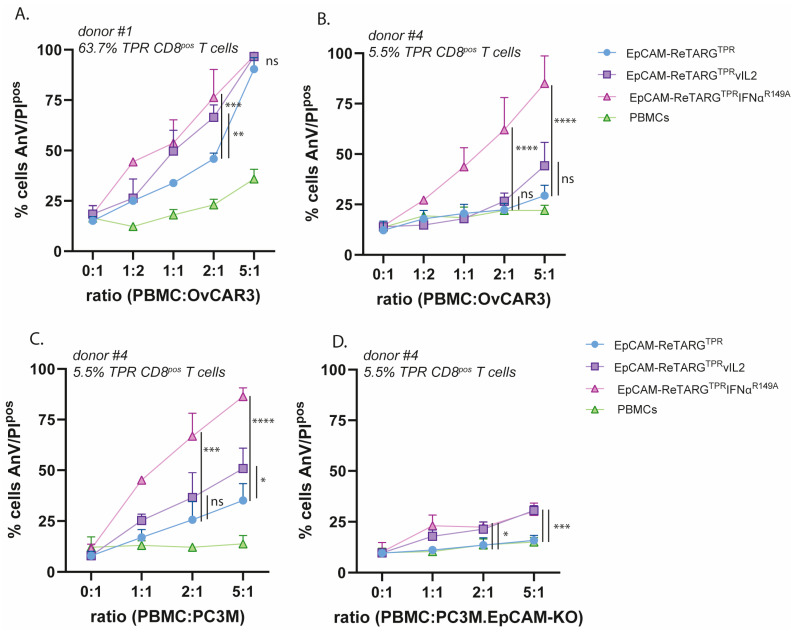
EpCAM-ReTARG^TPR^IFNα^R149A^ potently enhances the cytotoxic capacity of ex vivo-expanded TPR-specific CD8^pos^ T cells and retains EpCAM-selective activity. (**A**,**B**) The cytotoxic capacity of TPR/IL2-expanded PBMCs of donor #1 (**A**) and donor #4 (**B**) towards EpCAM^pos^ OvCAR3 cells at increasing PBMC/target cell ratios, treated with either EpCAM-ReTARG^TPR^, EpCAM-ReTARG^TPR^vIL2, or EpCAM-ReTARG^TPR^IFNα^R149A^ (all 10 nM). (**C**,**D**) The cytotoxic capacity of TPR/IL2-expanded PBMCs towards EpCAM^pos^ PC3M cells (**C**) and PC3M.EpCAM-KO cells (**D**) at increasing PBMC/target cell ratios, redirected by either EpCAM-ReTARG^TPR^, EpCAM-ReTARG^TPR^vIL2, or EpCAM-ReTARG^TPR^IFNα^R149A^ (all 10 nM). Apoptotic cancer cell death was assessed using Annexin-V/PI staining after 24 h. Statistical analysis was performed using one-way ANOVA. (ns  =  not significant, * *p* < 0.05, ** *p* < 0.01, *** *p* < 0.001, and **** *p* < 0.0001).

Both EpCAM-ReTARG^TPR^vIL2 and EpCAM-ReTARG^TPR^IFNα^R149A^ significantly enhanced the elimination of OvCAR3 cells by PBMCs from donor #1 (PBMC:OvCAR3 cell ratios of 1:1 and 2:1, respectively, (Figure 3A)). Notably, EpCAM-ReTARG^TPR^IFNα^R149A^ significantly enhanced the cytotoxic capacity of PBMCs of donor #4, whereas EpCAM-ReTARG^TPR^vIL2 only marginally enhanced tumor cell lysis even at the highest PBMC:OvCAR3 (5:1) cell ratio (Figure 3B). Next, we evaluated whether EpCAM-restricted activity for EpCAM-ReTARG^TPR^vIL2 and EpCAM-ReTARG^TPR^IFNα^R149A^ was maintained. To this end, parental PC3M versus PC3M-EpCAM-KO cancer cells were co-cultured at incremental PBMC/target cell ratios (donor #4) and treated with the respective fusion proteins. EpCAM-ReTARG^TPR^ induced ~35% lysis of target cells (5:1 PBMCPC3M cell ratio), which was enhanced by 15% and 56% upon treatment with EpCAM-ReTARG^TPR^vIL2 and EpCAM-ReTARG^TPR^IFNα^R149A, respectively^ (Figure 3C). Importantly, in analogous experiments using PC3M.EpCAM-KO cells, EpCAM-ReTARG^TPR^vIL2 induced a similar increase in cancer cell lysis of ~15%, suggesting that EpCAM-ReTARG^TPR^vIL2 enhances cancer cell lysis irrespective of EpCAM expression. Consequently, EpCAM-ReTARG^TPR^vIL2 was excluded from further study from here on.

### 2.4. EpCAM-ReTARG^TPR^IFNα^R149A^ Has Direct Antiproliferative Activity Towards Cancer Cells and Simultaneously Promotes Anticancer Capacity, Cytokine Production, and the Viability of TPR-Specific CD8^pos^ T Cells

**Figure 4:**
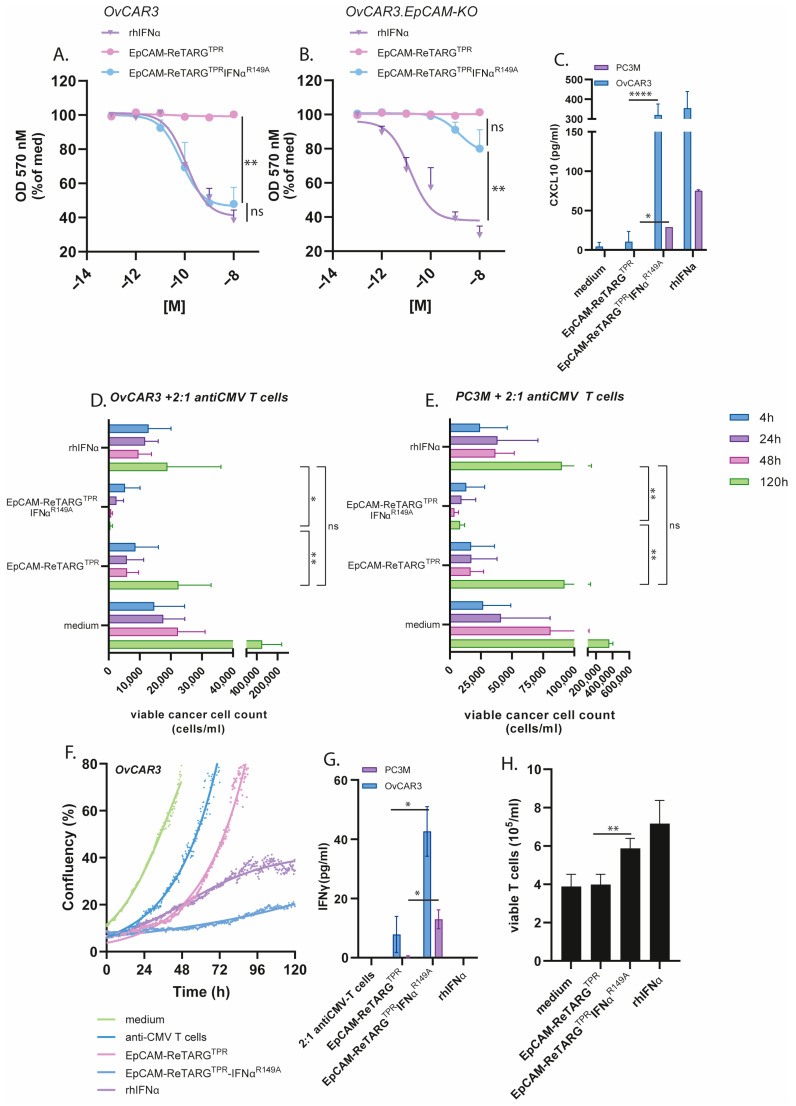
EpCAM-ReTARG^TPR^IFNα^R149A^ has direct antiproliferative activity towards cancer cells and simultaneously promotes anticancer capacity, cytokine production, and the viability of TPR-specific CD8^pos^ T cells. (**A**,**B**) (**A**) OvCAR3 or (**B**) OvCAR3.EpCAM-KO cancer cells treated for 8 d with increasing concentrations (0.0001- 10 nM) of EpCAM-ReTARG^TPR^, EpCAM-ReTARG^TPR^IFNα^R149A^, or rhIFNα. Medium values were set to 100%. (**C**) The capacity of EpCAM-ReTARG^TPR^ and EpCAM-ReTARG^TPR^IFNα^R149A^ to induce CXCL10 secretion by PC3M and OVCAR3 cells was quantified through ELISA. (D-E) The cytotoxic capacity of TPR/IL2-expanded PBMCs towards OvCAR3 (**D**) or PC3M (**E**) cancer cells treated with EpCAM-ReTARG^TPR^ and EpCAM-ReTARG^TPR^IFNα^R149A^ (both 10 nM) at a PBMC/target cell ratios  of 2:1. Apoptotic cancer cell death was assessed using Annexin-V/PI staining after 4, 24, 48, and 120 h. (**F**) The cell confluency of OvCAR3 cells treated with rhIFNα, EpCAM-ReTARG^TPR^, or EpCAM-ReTARG^TPR^IFNα^R149A^ in the co-culture with TPR/IL2-expanded PBMCs (PBMC/target cell ratio =  2:1) was measured with Phi Holomonitor™ for 120 h. (**G**) The capacity of EpCAM-ReTARG^TPR^ and EpCAM-ReTARG^TPR^IFNα^R149A^ to induce IFNγ secretion of TPR/IL2-expanded PBMCs in co-cultures with cancer cells was quantified through ELISA. (**H**) The capacity of EpCAM-ReTARG^TPR^ and EpCAM-ReTARG^TPR^IFNα^R149A^ to enhance the viability of PBMCs. Cell number was assessed through flow cytometry after 5 d. Graphs (**A**–**G**): *n*  =  3 (two technical replicates); means  ±  SDs are shown. Statistical analysis in A, B–D, and E was performed using one-way ANOVA. (ns  =  not significant, * *p* < 0.05, ** *p* < 0.01, and **** *p* < 0.0001). Statistical analysis in C, G, and H was performed using unpaired t-test (EpCAM-ReTARG^TPR^ versus EpCAM-ReTARG^TPR^IFNα^R149A^).

**Figure A5:**
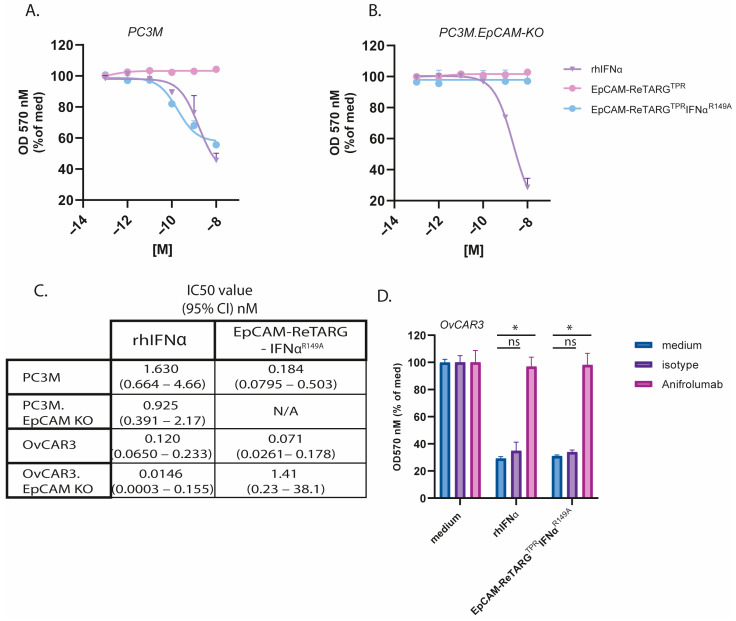
EpCAM-ReTARG^TPR^IFNα^R149A^ inhibits cancer cell proliferation in an EpCAM-restricted manner. (**A**,**B**) A proliferation assay of (**A**) PC3M or (**B**) PC3M.EpCAM-KO cancer cells treated with increasing concentrations (0.0001–10 nM) of EpCAM-ReTARG^TPR^, EpCAM-ReTARG^TPR^IFNα^R149A^, or IFNα-2b. Medium values were set to 100%. (**C**) Table presenting calculated IC50 values of assays of A, B, and Figure 4A/B (OvCAR3, OvCAR3 EpCAM-KO) in nM. (**D**) EpCAM-ReTARG^TPR^IFNα^R149A^- and IFNα-2b-induced growth inhibition of OvCAR3 cells was abrogated by incubation with a surplus of the IFNAR1-blocking antibody anifrolumab. Statistical analysis was performed using one-way ANOVA (ns  =  not significant, * *p* < 0.05).

The treatment of OvCAR3 and OvCAR3-EpCAM-KO cells with EpCAM-ReTARG^TPR^IFNα^R149A^ (0.0001–10 nM) in the absence of TPR-specific CD8^pos^ T cells for 8 d selectively inhibited cancer cell proliferation in both an EpCAM-restricted and dose-dependent manner, whereas similar treatment with EpCAM-ReTARG^TPR^ or rhIFNα failed to do so. This antiproliferative activity of EpCAM-ReTARG^TPR^IFNα^R149A^ was abrogated when treatment was performed in the presence of a surplus of the IFNAR1-blocking antibody anifrolumab (Figure 4A,B, and Figure A4). Analogous results were obtained when treating PC3M and PC3M-EpCAM-KO cells (Figure A5). It is reported that the IFNα treatment for cancer cells stimulates CXCL10 secretion, which recruits effector T cells to the tumor microenvironment [13]. Indeed, the EpCAM-ReTARG^TPR^IFNα^R149A^ treatment for both OvCAR3 and PC3M cells induced CXCL10 secretion (Figure 4C). Next, we compared EpCAM-ReTARG^TPR^IFNα^R149A^ and EpCAM-ReTARG^TPR^ for capacity to redirect enriched (90%) TPR-specific CD8^pos^ T cells to eliminate OvCAR3 cells in a co-culture experiment. After 24 h of co-culture, similar >70% cancer cell elimination was detected for EpCAM-ReTARG^TPR^IFNα^R149A^ and EpCAM-ReTARG^TPR^. However, after 120 h of treatment, anticancer activity induced by EpCAM-ReTARG^TPR^IFNα^R149A^ progressed up to 17-fold higher than that induced by EpCAM-ReTARG^TPR^ (Figure 4D,E). These results were corroborated by OvCAR3 cell confluency analysis through live-cell imaging (Figure 4F). Subsequently, we compared the immunomodulatory capacities of EpCAM-ReTARG^TPR^IFNα^R149A^, EpCAM-ReTARG^TPR^, and rhIFNα towards TPR-specific CD8^pos^ T cells. It was previously shown that the treatment of anti-CMV CD8^pos^ T cells with IFNα increases IFNγ production and can promote T-cell survival. We analyzed conditioned supernatants of our co-culture experiments and show that treatment with EpCAM-ReTARG^TPR^IFNα^R149A^ induced a 4-fold increase in IFNγ secretion (Figure 4G). In addition, we show that the treatment of TPR/IL2-expanded PBMCs with EpCAM-ReTARG^TPR^IFNα^R149A^ in the absence of cancer cells promotes T-cell viability and persistence and increases the number of viable T cells from 4 × 10^5^ up to 6 × 10^5^ cells/mL compared to treatment with EpCAM-ReTARG^TPR^ (Figure 4H). Together, these findings suggest that EpCAM-ReTARG^TPR^IFNα^R149A^ has direct antiproliferative activity towards cancer cells and simultaneously promotes anticancer capacity, cytokine production, and the viability of TPR-specific CD8^pos^ T cells.

### 2.5. EpCAM-ReTARG^TPR^IFNα^R149A^ Redirects TPR/IL2-Expanded PBMCs to Selectively Eliminate Primary Patient-Derived Merkel Cell Carcinoma Cells

**Figure 5:**
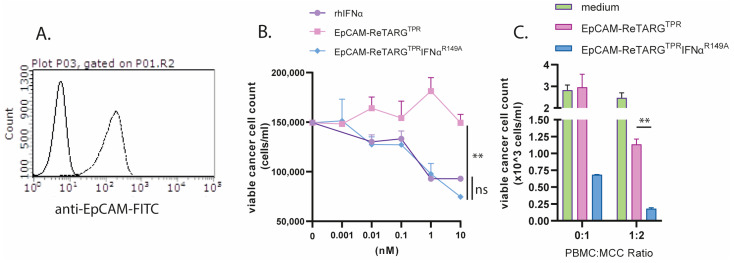
EpCAM-ReTARG^TPR^IFNα^R149A^ redirects TPR/IL2-expanded PBMCs to selectively eliminate primary patient-derived Merkel cell carcinoma cells. (**A**) Analysis of EpCAM expression by primary MCC cells via flow cytometry (**B**) Cytotoxic capacity of EpCAM-ReTARG^TPR^, EpCAM-ReTARG^TPR^IFNα^R149A^, and rhIFNα (0.001–10 nM) to reduce viable primary MCC cell number. (**C**) Capacity of EpCAM-ReTARG^TPR^ and EpCAM-ReTARG^TPR^IFNα^R149A^ to redirect TPR/IL2-expanded PBMCs (PBMC/target cell ratio = 1:2) to eliminate MCC cells. Cell number was analyzed after 3 d of incubation through flow cytometry. Graphs (**A**,**B**): *n*  =  1 (two technical replicates), means  ±  SEMs are shown. Statistical analysis was performed using one-way ANOVA (**B**) and unpaired t-test (**C**). (ns  =  not significant and ** *p* < 0.01).

Merkel cell carcinoma (MCC) is a notoriously refractory type of skin cancer that expresses EpCAM. Previously, it was reported that MCC shows sensitivity to treatment with IFNα [14,15]. We reasoned that immunotherapy with EpCAM-ReTARG^TPR^IFNα^R149A^ could be an attractive treatment option for this highly malignant cancer type. We confirmed EpCAM expression in cancer cells freshly derived from an MCC patient (Figure 5A). Subsequently, MCC cells were treated with a dose range of 0.001–10 nM of EpCAM-ReTARG^TPR^, EpCAM-ReTARG^TPR^IFNα^R149A^, and rhIFNα, respectively, which reduced cancer cell viability by half (Figure 5B). When co-cultured with TPR/IL2-expanded PBMCs, EpCAM-ReTARG^TPR^IFNα^R149A^ induced a marked 3-fold reduction in MCC cell number compared to EpCAM-ReTARG^TPR^ (Figure 5C).

## 3. Discussion

Previously, we reported on a novel Fab–peptide–HLA-I fusion protein, designated EpCAM-ReTARG^TPR^, that consists of an EpCAM-directed Fab antibody fragment fused to HLA-B*07:02-β2M that is genetically equipped with the CMV pp65-derived peptide TPR. EpCAM-ReTARG^TPR^ showed potent in vitro capacity to redirect anti-CMV_pp65_ CD8^pos^ T cells from CMV-seropositive/HLA-B*07:02^pos^ donors to selectively eliminate various EpCAM-expressing carcinoma cell lines and primary patient-derived cancer cells [4]. Building upon these results, we aimed to further enhance and broaden its anticancer activities by fusing it to the immune-potentiating cytokines vIL2 and IFNα^R149A^, respectively. EpCAM-ReTARG^TPR^vIL2 was equipped with two IL2^H16A, F42A^ mutein (vIL2) molecules; we hypothesize that this would selectively promote the cytotoxic activity of redirected TPR-specific CD8^pos^ T cells. However, compared to EpCAM-ReTARG^TPR^, this showed only marginal enhanced capacity to induce cancer cell lysis. Moreover, EpCAM-selective anticancer activity of EpCAM-ReTARG^TPR^vIL2 appeared to be strongly compromised. Consequently, we decided to exclude EpCAM-ReTARG^TPR^vIL2 from our study. 

Our results appear to contradict with those of Schardt et al., who reported increased cytotoxicity and the expansion of anti-CMV_pp65_ CD8^pos^ T cells directed to EGFR^pos^ tumor cells when using an analogous guided-pMHC-staging (GPS) molecule that was equipped with the same IL2 mutein (H16A and F42A) [16]. However, their experimental set-up of cytotoxicity assays may be incomplete since the authors did not include control experiments using cancer cells in which EGFR expression was knocked down/out.

We equipped EpCAM-ReTARG^TPR^IFNα^R149A^ with one IFNα^R149A^ mutein unit, which was reported to have a 200-fold reduced binding affinity for IFNAR2. Intriguingly, EpCAM-ReTARG^TPR^IFNα^R149A^ potently enhanced the cytotoxic capacity of TPR/IL2-expanded PBMCs. Notably, unlike EpCAM-ReTARG^TPR^vIL2, EpCAM-ReTARG^TPR^IFNα^R149A^-enhanced cytotoxicity remained fully EpCAM-restricted. Interestingly, EpCAM-ReTARG ^TPR^IFNα^R149A^ enhanced cancer cell lysis when PBMCs derived from donor #4 were used, who had a low percentage of TPR CD8^pos^ T cells compared to donor #1. This indicates that EpCAM-ReTARG^TPR^IFNα^R149A^ may augment the cytotoxic abilities of not only specific TPR CD8^pos^ T cells but also other immune cells within the PBMC population, which could be particularly advantageous for individuals with a low proportion of TPR-specific cells. In 120 h target/effector cell co-culture experiments, EpCAM-ReTARG^TPR^IFNα^R149A^ significantly reduced the viable cell number of EpCAM^pos^ cancer cells compared to EpCAM-ReTARG^TPR^.

It appears reasonable to assume that the unique and multifold anticancer activities we observed in our co-culture experiments with EpCAM-ReTARG^TPR^IFNα^R149A^ are in part attributable to the pleiotropic and possibly mutually reinforcing biological activities of its IFNα^R149A^ domain towards the targeted cancer cells as well as the engaged TPR-specific CD8^pos^ T cells.

In this respect, in the absence of TPR-specific CD8^pos^ T cells, the treatment of OvCAR3 and PC3M cancer cells with EpCAM-ReTARG^TPR^IFNα^R149A^ inhibits proliferation and stimulates cancer cell-secreted CXCL10 (IP-10). CXCL10 is a chemoattractant for activated T cells and may enhance the homing of T cells to the tumor site(s) [13]. Additionally, in co-culture experiments, treatment with EpCAM-ReTARG^TPR^IFNα^R149A^ boosted T-cell-secreted IFNγ, and we observed that treatment with EpCAM-ReTARG^TPR^IFNα^R149A^ prolonged the survival of TPR/IL2-expanded PBMCs. Together, these characteristics enhance the anticancer activity of EpCAM-ReTARG^TPR^IFNα^R149A^, leading to improved cancer cell lysis, which corroborates findings by Hervas-Stubbs et al., who showed that treatment with IFNα during the in vitro expansion of anti-CMV CD8^pos^ T cells increases IFNγ production and enhances their cytolytic capacity [17].

Pogue et al. demonstrated that an anti-CD38 antibody genetically equipped with an attenuated version of human IFNα (designated anti-CD38-IFNα(att)) exhibits 10,000-fold increased specificity for CD38^pos^ cells in vitro compared to non-targeted IFNα. As a result, anti-CD38-IFNα(att) is approximately 6000-fold less toxic to normal bone marrow cells in vitro than IFNα [18]. Although anti-CD38-IFNα(att) provided potent anti-tumor activity in various MM cell lines and in human xenograft MM tumor models, it is not addressed whether it induces T cell/immune cell activation. In a study by Daneels et al., the in vivo antitumor potential of the huCD20-IFNαR149A fusion protein (huCD20-Fc-AFN) was explored using tumor-bearing human immune system (HIS) mice. huCD20-Fc-AFN not only directly affected cancer cell growth but also significantly enhanced immune cell-mediated tumor cell elimination. Upon huCD8^pos^ T-cell depletion, tumor growth was significantly increased, suggesting a crucial role of T cells in cancer cell elimination [19]. Future studies aimed to evaluate the in vivo anti-tumor activity of EpCAM-ReTARG^TPR^IFNα^R149A^ should use tumor-bearing mice engrafted with peripheral blood mononuclear cells (PBMCs) from CMV-seropositive/HLA-B*07:02-matched donors.

Merkel cell carcinoma (MCC) is a rare EpCAM-expressing refractory skin cancer. It has been reported that MCC is sensitive to treatment with IFNα [15]. Therefore, we reasoned that EpCAM-ReTARG^TPR^IFNα^R149A^ could be an attractive treatment option for this cancer type. Indeed, our results show that MCC cells were sensitive to EpCAM-ReTARG^TPR^IFNα^R149A^-induced growth inhibition, and they were more effectively eliminated by TPR/IL2-expanded PBMCs compared to EpCAM-ReTARG^TPR^. Recent studies have shown that Merkel cell polyomavirus (MCPyV) plays a vital role in the development of MCC by encoding oncoproteins. Interestingly, T cells targeting MCPyV oncogenes, specifically large T (LTA) and small (STA) antigens, are found exclusively in MCC patients, and these LTA- and STA-specific T cells can effectively kill HLA-matched MCPyV-positive MCC cells [20]. Given the sensitivity of MCC cells to IFNα, developing IFNα-equipped Fab–peptide–HLA-I fusion proteins equipped with MCPyV-derived epitopes may represent a promising new immunotherapy approach for MCPyV-associated MCC.

Recombinant untargeted IFNα is currently used in the treatment of several cancers, including melanoma, hairy cell leukemia, and renal cell carcinoma (RCC) [21]. Notably, a study by Rosato et al. [2] found that anti-CMV T cells infiltrate RCC tumors and can be activated by viral peptides, suggesting that EpCAM-ReTARG^TPR^IFNα^R149A^ could be particularly effective for RCC treatment. Additionally, other IFNα-sensitive EpCAM^pos^ cancer types may also be promising targets for EpCAM-ReTARG^TPR^IFNα^R149A^ therapy.

Decoration of of EpCAM^pos^ cancer cells with EpCAM-ReTARG^TPR^ enables physiological engagement of cognate anti-CMV CD8^pos^ T cells and subsequent effective target cell elimination in the absence of excessive cytokine release. Compared to an analogous BiTE, we hypothesize that EpCAM-ReTARG^TPR^IFNα^R149A^ is less likely to induce severe side effects such as cytokine release syndrome. However, future in vivo studies should be conducted to directly compare the respective anticancer efficacies and cytokine secretion profiles. It is important to note that EpCAM is expressed at low levels on the basolateral side of epithelial cells, which may result in off-target effects. Truly cancer-selective targets are rare, but if identified, the ReTARG fusion protein can be readily adapted to target them.

In conclusion, the armoring of the carcinoma-directed peptide–HLA-I fusion protein EpCAM-ReTARG^TPR^ with IFNα^R149A^ potently enhanced the efficacy of pre-existing anti-CMV CD8^pos^ T-cell immunity to selectively eliminate EpCAM^pos^ cancer cells.

## 4. Materials and Methods

### 4.1. Antibodies and Reagents

The following primary (fluorescent)-labeled monoclonal antibodies directed against human antigens were used: FITC-labeled anti-EpCAM (clone VU-1D9, STEMCELL Technologies Germany GmbH, Köln, Germany, APC-labeled anti-HLA-B7 (clone BB7.1), BioLegend Europe B.V., Amsterdam, The Netherlands, APC-labeled anti-IL2 (clone MQI-17H12, and CaptureSelect™ Biotin Anti-IgG-CH1 conjugate (were from Thermo Fisher Scientific, Waltham, MA, USA). Anti-human IFNAR1 (anifrolumab) #SIM0022 was from BioXCell, Lebanon, NH, USA. The neutralizing IL2 antibody (clone 5334) was from R&D Systems, Inc., Minneapolis, NE, USA. The following reagents were used: trypan blue (Sigma Aldrich, Zwijndrecht, The Netherlands), Streptavidin-AlexaFluor™647 (Thermo Fisher Scientific, Waltham, MA USA), FITC-labeled Annexin-V (ImmunoTools GmbH, Friesoythe, Germany), and propidium iodide (PI) (Thermo Fisher Scientific, Waltham, MA USA). The following recombinant proteins were used: human IFNα-2b and human IL2. They were from ImmunoTools GmbH, Friesoythe, Germany. Biotinylated MHC I-Strep HLA-B*0702, CMV pp65 (TPRVTGGGAM), and APC-labeled strep-Tactin were from IBA Lifesciences GmbH, Göttingen, Germany.

### 4.2. Cell Lines and Transfectants

The cell lines Jurkat (T-ALL), CTLL2 (mouse cytotoxic T-cell clone), PC3M (prostate cancer), and OvCAR3 (ovarian cancer) were obtained from the ATCC (Manassas, VA, USA). PC3M.EpCAM-KO and OvCAR3.EpCAM-KO cells were generated using CRISPR-Cas9 gene editing technology via transfection with the plasmid pSpCas9 BB-2A-GFP (PX458) containing sgRNA 5′-TAATGTTATCACTATTGATC-3′ [13]. Subsequently, EpCAM-KO cancer cells were obtained through limited dilution. OvCAR3.pp65 cells were generated via lipofection (Fugene-HD, Promega BNL, Leiden, The Netherlands) of the plasmid pCMV6-pp65 (OriGene Technologies GmbH, Herford, Germany), and OvCAR3.pp65 cells stably expressing the CMV pp65 protein were obtained after limited dilution. Cells were cultured in RPMI-1640 or DMEM (Lonza, Geleen, The Netherlands) supplemented with 10% FCS at 37° C in a humidified 5% CO_2_ atmosphere.

### 4.3. Merkel Cell Carcinoma Patient Sample

Merkel cell carcinoma tissues was obtained from surgical resection waste materials. Tumor tissue was minced and short-term cultured in RPMI/10% fetal calf serum. Cell phenotype was analyzed through flow cytometry (Guava Easycyte, Merck millipore, Amsterdam, The Netherlands) using fluorescently labeled antibodies. The study was conducted in accordance with the Declaration of Helsinki, and the protocol was approved by the medical ethical committee of the UMCG, under approval number EUCTR2012-000507-33-EN on 26 November 2012.

### 4.4. Assessment of Cytokine Activity of EpCAM-ReTARG^TPR^vIL2

The presence of the vIL2 domains in EpCAM-ReTARG^TPR^vIL2 was confirmed using flow cytometry. In short, EpCAM^pos^ cancer cells were incubated with EpCAM-ReTARG^TPR^ or EpCAM-ReTARG^TPR^vIL2. Subsequently, cancer cell surface-bounded vIL2 was assessed using an APC-labeled anti-IL2 antibody and an IL2-neutralizing antibody clone 5334 (R&D Systems, Inc., Minneapolis, NE, USA) with a secondary anti-mouse-647 antibody (Thermo Fisher Scientific, Waltham, MA USA). The T-cell proliferative activity of EpCAM-ReTARG^TPR^vIL2 was assessed using the IL2-dependent mouse cell line CTLL2. In short, CTLL2 cells were cultured in the presence of increasing concentrations of EpCAM-ReTARG^TPR^vIL2 or EpCAM-ReTARG^TPR^ (0.078–20 nM). IL2-mediated proliferation of cells was determined after 72 h using flow cytometry. Moreover, EpCAM-ReTARG^TPR^vIL2-induced CTLL2 proliferation was assessed in the presence of an IL2-neutralizing antibody.

### 4.5. Assessment of Cytokine Activity of EpCAM-ReTARG^TPR^IFNα^R149A^

OvCAR3, OvCAR3.EpCAM-KO, and Jurkat cells were transduced with the interferon-stimulated response element (ISRE) luciferase reporter lentivirus (BPS Bioscience, San Diego, CA, USA #79824) at MOIs of 100, 100, 10, respectively. After 72 h, transduced cells were selected from the culture medium supplemented with 1.0 µg/mL puromycin. Subsequently, ISRE luc induction by IFNα was assessed. In short, OvCAR3 cells and OvCAR3.EpCAM-KO ISRE reporter cells were seeded (each 8000 cells/well) using 96-well black/clear bottom plates (ThermoFisher) and were left to adhere overnight. Cells were treated with increasing concentrations (0.0001–10 nM) of IFNα-2b, EpCAM-ReTARG^TPR^, and EpCAM-ReTARG^TPR^IFNα^R149A^ for 6 h. After treatment, the Bio-Glo™ luciferase reagent (Promega BNL, Leiden, The Netherlands)) was added to each well, and luminescence was measured using a SpectraMaxi3X molecular device. Analogously, a suspension of 50,000 Jurkat.ISRE-luc reporter cells was added to 96-wells black/clear bottom plates, and cells were treated and evaluated as described above.

### 4.6. Ex Vivo Expansion of PBMCs from HLA-B*07:02^pos^/CMV-Seropositive Individuals

Peripheral blood mononuclear cells (PBMCs) from HLA-B*07:02^pos^/CMV-seropositive individuals were purchased from CTL - Europe GmbH, Rutesheim Germany PBMCs were harvested, washed, and cultured in 6-well plates (a final concentration of 4 × 10^6^ cells/mL, with 2 mL per well) in the RPMI medium. PBMCs were stimulated with 0.5 µg/mL CMV pp65-derived peptide TPR for 4 d. Next, TPR-stimulated PBMCs were harvested, resuspended in a fresh X-VIVO15 medium (Lonza, Geleen, The Netherlands) supplemented with 50 IU/mL IL2, and cultured for an additional 7 d. The percentage of TPR-specific HLA-B*07:02^pos^ CD8^pos^ T cells was determined using streptamer staining through flow cytometry.

### 4.7. Construction, Production, and Purification of EpCAM-ReTARG^TPR^, EpCAM-ReTARG^TPR^vIL2, and EpCAM-ReTARG^TPR^IFNα^R149A^

EpCAM-ReTARG^TPR^ was designed as a monomeric recombinant fusion protein consisting of the antigenic CMV pp65 peptide TPR, β2M, and a truncated HLA-B*07:02 heavy chain lacking the transmembrane and intracellular domains. To enhance the stability of EpCAM-ReTARG^TPR^, The C-terminus of TPR was fused to the flexible linker sequence G**C**GGSGGGGSGGGGS, which was engineered to contain a cysteine residue (in bold) that promotes the formation of a stabilizing intramolecular disulfide bridge with a cysteine residue inserted in the α1 domain HLA-B*07:02 heavy chain. Using a flexible linker, the HLA-I α chain was genetically fused to a high-affinity anti-EpCAM Fab antibody fragment containing the VH-VL gene segments of the humanized scFv 4D5 MOC-B [14]. In this Fab, we employed human kappa CL and CH1 domains based on UniProt accession numbers P01834 and P01857, respectively. To construct EpCAM-ReTARG^TPR^vIL2, two mutein IL2 (vIL2) molecules containing the point mutations, F42A and H16A, were genetically fused to the C-terminal of the constant domain of the light chain (CL) of the anti-EpCAM Fab antibody of EpCAM-ReTARG^TPR^. To construct EpCAM-ReTARG^TPR^IFNα^R149A^, an IFNα-2b-encoding DNA fragment with the R149A point mutation was genetically fused to the CL domain of the anti-EpCAM Fab antibody of EpCAM-ReTARG^TPR^. The amino acid sequence of mutein IL2^H16A/F42A^ was obtained from a study by Quayle et al. [8], and the amino acid sequence of mutein IFNα^R149A^ was obtained from Patent number US20180028616A1. The cDNAs encoding the respective fusion proteins were synthesized and then cloned into the eukaryotic expression plasmid pcDNA3.1-hygro by Genscript (Rijswijk, The Netherlands) and then transfected (Fugene-HD, Promega) into Hek293AD production cells. After 7 d, conditioned cell culture supernatants were harvested and cleared via centrifugation (4000× *g*, 30 min). EpCAM-ReTARG^TPR^, EpCAM-ReTARG^TPR^vIL2, and EpCAM-ReTARG^TPR^IFNα^R149A^ were purified using a Capture Select™ CH1-XL column (Thermo Fisher Scientific, Waltham, MA, USA) connected to an ÄKTA Start chromatography system (GE Healthcare Life Sciences, Eindhoven, The Netherlands), diluted in PBS to 1 mg/mL, and stored at −20 °C until use.

### 4.8. SDS-PAGE Analysis

Purified EpCAM-ReTARG^TPR^, EpCAM-ReTARG^TPR^vIL2, and EpCAM-ReTARG ^TPR^IFNα^R149A^ (2 μg protein per lane) were separated using SDS-PAGE (10% acrylamide) under reducing or non-reducing conditions and stained using Coomassie brilliant blue.

### 4.9. Assessment of EpCAM-Binding Activity of EpCAM-ReTARG^TPR^, EpCAM-ReTARG^TPR^vIL2, and EpCAM-ReTARG^TPR^IFNα^R149A^

The binding of EpCAM-ReTARG^TPR^, EpCAM-ReTARG^TPR^vIL2, and EpCAM-ReTARG^TPR^IFNα^R149A^ to cell surface-expressed EpCAM was assessed through flow cytometry. In short, EpCAM^pos^- and EpCAM-KO-derived PC3M cancer cells were incubated with 10 nM of either EpCAM-ReTARG^TPR^, EpCAM-ReTARG^TPR^vIL2, or EpCAM-ReTARG ^TPR^IFNα^R149A^ at 4° C for 45 min, after which binding was evaluated using CaptureSelect™ Biotin Anti-IgG-CH1 Conjugate plus Streptavidin-AlexaFluor™647 (Thermo Fisher Scientific, Waltham, MA USA) or the fluorescently labeled anti-HLA-B7 antibody.

### 4.10. Cancer Cell Proliferation Assay

To evaluate the antiproliferative activity of EpCAM-ReTARG^TPR^IFNα^R149A^, EpCAM-expressing cancer cells (or EpCAM-KO derivatives) were seeded in 48-well plates at 3500 cells/well. Cells were treated with 0.001–10 nM of IFNα-2b, EpCAM-ReTARG^TPR^, or EpCAM-ReTARG^TPR^IFNα^R149A^ for 8 d. Cancer cell proliferation was quantified via the OD of crystal violet staining measured at 570 nm using a microplate reader (SpectraMaxi3X, Molecular Devices, San Jose, CA, USA). Additionally, the antiproliferative activity of EpCAM-ReTARG^TPR^IFNα^R149A^ towards cancer cells was assessed in the presence of the IFNAR1-blocking antibody anifrolumab. The IC50 was determined using a colorimetric crystal violet assay, a standard assay to evaluate cell viability, cell attachment, and cell proliferation. OD values from this assay were normalized to the percentage of the medium control (set to 100%). The concentrations of the EpCAM-ReTARG^TPR^ fusion protein variants were transformed to the logarithm of the dose and plotted using the GraphPad Prism software, version 10.2.3. IC50 values, along with 95% confidence intervals, were calculated by performing a non-linear regression analysis using the “log(inhibitor) vs. response (three parameters)” equation. This method follows the guidelines outlined in the *GraphPad Prism 10 Curve Fitting Guide* for determining absolute IC50 values. A reference to this procedure is included in the revised manuscript.

### 4.11. Viability Assay

Ex vivo TPR/IL2-expanded PBMCs were seeded in a 48-well plate (400,000 cells/well) and cultured in the presence of IFNα-2b (referred to as rhIFNα), EpCAM-ReTARG^TPR^, or EpCAM-ReTARG^TPR^IFNα^R149A^, respectively, for 120 h. The number of viable PBMCs was evaluated using flow cytometry (PI staining).

### 4.12. In Vitro Cytotoxicity Assays

EpCAM^pos^ cancer cells (or EpCAM-KO derivatives) were treated with either EpCAM-ReTARG^TPR^, EpCAM-ReTARG^TPR^vIL2, or EpCAM-ReTARG^TPR^IFNα^R149A^ in the presence (or absence) of ex vivo-expanded PBMCs at the indicated PBMC-to-target cell ratios. Apoptotic cancer cell death was evaluated using flow cytometry at indicated timepoints (Annexin V/PI staining). In addition, the anticancer activity of EpCAM-ReTARG^TPR^IFNα^R149A^ was assessed through live-cell imaging using a Phi HoloMonitor™ system (Phase Holographic Imaging PHI AB, Lund, Sweden) as the mean of cell confluency after 120 h.

### 4.13. Assessment of Cytokine Secretion

The activation of TPR/IL2-expanded PBMCs in response to treatment with EpCAM-ReTARG^TPR^ and EpCAM-ReTARG^TPR^-IFNα^R149A^ (10 nM each), respectively, was determined by co-culturing PBMCs with cancer cells at the indicated PBMC-to-target cell ratios. The conditioned culture media was collected after treatment for 72 h, after which the levels of human IFNγ were determined via ELISA (ThermoFisher). In addition, we assessed CXCL10 secretion by cancer cells after treatment for 72 h with rhIFNα, EpCAM-ReTARG^TPR^, and EpCAM-ReTARG^TPR^IFNα^R149A^ (10 nM each), respectively, and assessed conditioned supernatants for CXCL10 secretion using a corresponding ELISA (ThermoFisher).

### 4.14. Statistical Analysis

Statistical analyses were performed using GraphPad Prism 8 (GraphPad Software, version 10.2.3). Means of differences were calculated using one- and two-way ANOVA, respectively, followed by a multiple comparison test where appropriate. *p*-values considered significant are indicated by asterisks as follows: * *p* < 0.05; ** *p* < 0.01; *** *p* < 0.001.

## Data Availability

All data relevant to this study are included in the article.

## Abbreviations

The following abbreviations are used in this manuscript:

β2M	Beta-2-Microglobulin
CL	Constant domain of the light chain
CMV	Cytomegalovirus
GPS	Guided-pMHC-staging
HIS	Human immune system
IFNAR	Interferon alpha/beta receptor
IFNα	Interferon alpha
IL2	Interleukin 2
IL2R	Interleukin 2 receptors
ISRE	Interferon-stimulated response element
LTA	Large T-cell antigen
MCC	Merkel cell carcinoma
MCPyV	Merkel cell polyomavirus
MW	Molecular weight
PBMCs	Peripheral blood mononuclear cells
RCC	Renal cell carcinoma
STA	Small T-cell antigen
TPR	TPRVTGGGAM
T_reg_	Regulatory T cells
